# Association between Adiponectin Gene Polymorphism and Environmental Risk Factors of Type 2 Diabetes Mellitus among the Chinese Population in Hohhot

**DOI:** 10.1155/2020/6383906

**Published:** 2020-06-21

**Authors:** Meng Cui, Yumin Gao, Yanping Zhao, Hui Pang, Le Chen, Zhidi Wang, Lingyan Zhao, Minhui Li

**Affiliations:** ^1^Inner Mongolia Medical University, School of Public Health, Molecular Epidemiology Laboratory of Chronic Diseases, Hohhot Inner Mongolia 010110, China; ^2^Inner Mongolia Medical University, School of Public Health, Department of Epidemiology, Hohhot, Inner Mongolia 010110, China; ^3^Neurology Department, The Affiliated Hospital of Inner Mongolia Medical University, Hohhot, Inner Mongolia 010010, China; ^4^Inner Mongolia Institute of Traditional Chinese Medicine, Hohhot, Inner Mongolia 010020, China

## Abstract

**Objective:**

The aim of this study was to investigate the association between adiponectin gene polymorphisms *rs10937273*, *rs1501299*, *rs182052*, *rs2241767*, and *rs266729* and environmental risk factors of type 2 diabetes mellitus (T2DM) in Hohhot. The study explored different models of gene-environment interactions, aimed at providing approaches for the prevention and control of T2DM in combination with the characteristics of the local population.

**Methods:**

A case-control study was conducted including 406 Chinese participants, comprising 203 cases and 203 controls from various hospitals. Adiponectin (*ADIPOQ*) gene polymorphisms *rs10937273*, *rs1501299*, *rs182052*, *rs2241767*, and *rs266729* were detected using an improved multiple ligation detection reaction technique. Generalized multifactor dimensionality reduction (GMDR) and logistic regression were conducted to analyze the associations between adiponectin gene polymorphisms and T2DM, as well as the interactions between adiponectin gene polymorphisms and environmental factors.

**Results:**

*ADIPOQ* gene polymorphisms *rs10937273*, *rs1501299*, *rs182052*, *rs2241767*, and *rs266729* were associated with type 2 diabetes. Based on the haplotype of the five adiponectin gene single-nucleotide polymorphism (SNP) loci, we found that G-G-A-A-C was a susceptible haplotype of T2DM (*P* < 0.05). Interaction analyses demonstrated associations between *rs1501299* and central obesity (consistency = 80%, *P* = 0.011) and between *rs266729* and *rs182052* and central obesity (consistency = 70%, *P* = 0.011).

**Conclusions:**

Our findings indicate that there is an interaction between the *ADIPOQ* gene and central obesity, which provides new insights into the prevention and treatment of T2DM.

## 1. Introduction

Type 2 diabetes mellitus (T2DM) is a growing global public health concern; according to the latest statistics of the International Diabetes Federation (IDF) from 2019, there are 463 million people aged 20–79 with confirmed or undiagnosed diabetes globally, with 116 million individuals in China. The prevalence of diabetes mellitus has been increasing worldwide in recent decades owing to urbanization, changes in nutrition intake, obesity, and low exercise. Diabetes mellitus has become a chronic noncommunicable disease that seriously endangers public health [1]. In 2013, the overall prevalence of diabetes in Chinese adults was 10.9% [2], while the prevalence of impaired glucose tolerance was 35.7%. From 2000 to 2016, the number of diabetes cases in China increased by 62.7% [3]. The diabetes epidemic in China is very severe and diabetes prevention and treatment face many challenges, including poor diagnosis, a lack of data regarding basic health parameters or risk factors, a high misdiagnosis rate, and increased prevalence among younger individuals.

T2DM is caused by a combination of genetic and environmental factors. In recent years, several domestic and international epidemiological studies on T2DM risk factors have been conducted. Environmental risk factors of diabetes include age, obesity, dietary habits, physical activity, socioeconomic status, hypertension, and impaired glucose tolerance [4]. Genetic factors can also constitute potential risk factors of diabetes; individuals with genetic susceptibility are more likely to develop T2DM under the influence of environmental risk factors. Multiple genetic polymorphisms have been shown to be associated with genetic susceptibility to T2DM.

Adiponectin, an adipose tissue-derived bioactive protein (~30 kDa), possesses anti-inflammatory, antiatherosclerotic, antidiabetic, and insulin-sensitizing properties [5]. It is encoded by the adiponectin gene (*ADIPOQ*), which is located in chromosome 3q27.

As the possible associations between adiponectin single-nucleotide polymorphisms (SNPs) and environmental factors have not been extensively studied, we analyzed the polymorphisms of five adiponectin gene loci (*rs10937273*, *rs1501299*, *rs182052*, *rs2241767*, and *rs266729*) and investigated their gene-environment interactions.

## 2. Materials and Methods

### 2.1. Sample and Data Collection

A total of 406 unrelated individuals were included in this study: 203 T2DM patients from the Affiliated Hospital of Inner Mongolia Medical University, Inner Mongolia Autonomous Region Hospital of Traditional Chinese Medicine, and Inner Mongolia International Mongolian Medical Hospital and 203 healthy controls recruited from the Physical Examination Center of the Affiliated Hospital of Inner Mongolia Medical University.

The case group and the control group were all Han individuals. All participants provided signed informed consent.

#### 2.1.1. Inclusion Criteria

The participants were divided into a case and control group according to the 1999 World Health Organization (WHO) criteria for the diagnosis of T2DM [6]:

- Age ranges from 30 to 70 years
- The number of men and women is balanced

#### 2.1.2. Exclusion Criteria

- Patients with elevated blood glucose caused by other factors such as liver disease, acute infection under stress, the use of drugs affecting glucose metabolism, and endocrine diseases
- Patients with serious heart, lung, liver, kidney, and brain complications and other serious primary diseases
- Pregnant or lactating women
- Patients with acute metabolic disorders, such as diabetic ketoacidosis, during the past month

On the day of the physical examination (7:00 to 9:00 am), 5 mL of overnight fasting venous blood were collected in an anticoagulation ethylenediaminetetraacetic acid (EDTA) vacuum tube and a separating gel accelerating tube. Following centrifugation, the serum, plasma, and blood cells were separately collected in 2 mL cryotubes.

Case and control medical records/physical examination data, including weight, blood pressure, blood sugar, and blood lipids, were collected. Health questionnaires were designed and health surveys were conducted in both the case and control groups. These included basic demographic situation, environmental behavior, family history of T2DM, and body mass index (BMI). We defined current alcohol consumption as drinking within the past 30 days, with >6 standard drinking units (60 g alcohol) after conversion. Current smokers were defined as individuals who had smoked every day for more than three months. Central obesity was defined as a waist − to − hip ratio (WHR) > 0.9 for men and WHR > 0.8 for women; 24 kg/m^2^ ≤ BMI < 28 kg/m^2^ was considered overweight and BMI ≥ 28 kg/m^2^ indicated obesity. High-fat diet was defined as eating red meat more than twice a day on average, with a total oil intake per day of >30 g including frying and other high-calorie cooking methods [7]. Standard physical exercise was defined as continuous aerobic exercise for >30 min, at least five times a week.

### 2.2. DNA Extraction and Determination of Adiponectin Content

Genomic DNA was extracted from EDTA whole blood using a centrifugal column type whole blood genomic DNA rapid extraction kit, centrifugal column type (Blood Genome DNA Extraction Kit; TIANGEN Bio Company, Beijing, China). DNA concentration was determined using ultraviolet-visible spectrophotometry (NanoDrop 2000c Spectrophotometer; Thermo Fisher Scientific, Wilmington, DE, USA) and ranged from 18 to 90 ng/L.

Adiponectin serum levels were detected by enzyme-linked immunosorbent assay (ELISA) with a rabbit anti-human polyclonal antibody (bs-0471R, Abcam, Cambridge, MA, USA) using a standard curve (*R*^2^ > 0.99). Absorbance was measured using an Enzyme analyzer (Infinite F50, Tecan, Switzerland) at a wavelength of 450 nm. The ELISA was conducted by SinoBest Bio (Shanghai, China).

### 2.3. Genotyping

Single-nucleotide polymorphism genotyping was carried out using the improved multiple ligation detection reaction (IMLDR) technique developed by Genesky Biotechnologies Inc. (Shanghai, China). The target SNP locus was amplified in a single system by multiplex PCR. The amplified product was purified with an exonuclease and shrimp alkaline enzyme (ExoI/SAP) and used as the template for the subsequent ligase reaction. Alleles were identified using various specific fluorescently labeled nucleotide probes. Different SNPs were further distinguished by different extension lengths at the 3′ end. The connection products were identified by capillary electrophoresis using an ABI3730XL sequencer (Thermo Fisher Scientific, Wilmington, DE, USA).

Briefly, 1 *μ*L of the 1% DNA sample was used for quality inspection and concentration estimation. The sample was then diluted to a working concentration of 5–10 ng/*μ*L according to the estimated concentration. The multiplex PCR reaction system (20 *μ*L) contained 1× HotStarTaq buffer, 3.0 mM Mg^2+^, 0.3 mM dNTP, 1 U HotStarTaq polymerase (Qiagen Inc., Valencia, CA, USA), 1 *μ*L of the sample DNA, and 1 *μ*L of the multiplex PCR primers (Table 1). The PCR cycle program for the five SNPs was as follows: initial denaturation at 95°C for 2 min; 11 cycles of 94°C for 20 s, 65°C for 40 s, and 72°C for 1.5 min; 24 cycles of 94°C for 20 s, 59°C for 30 s, and 72°C for 1.5 min; and a final extension at 72°C for 2 min. The multiplex PCR products were then purified by adding 5 U of SAP enzyme and 2 U of exonuclease I enzyme to 10 *μ*L of PCR product, incubating the reaction at 37°C for 1 h, and then inactivating the reaction at 75°C for 15 min. The ligation reaction system included 10 *μ*L of ligation buffer, 0.25 *μ*L of high temperature ligase, 0.4 *μ*L of the 5′ ligation primer (1 *μ*M), 0.4 *μ*L of the 3′ ligation primer (2 *μ*M), 2 *μ*L of the purified multiplex PCR product, and 6 *μ*L of ddH_2_O. Finally, 0.5 *μ*L of the diluted ligation product was mixed with 0.5 *μ*L of Liz500 size standard and 9 *μ*L of Hi-Di, denatured at 95°C for 5 min, and then run on an ABI3730XL sequencer. Raw data were analyzed using GeneMapper 4.1 (Applied Biosystems, USA). All primers, probes, and labeling oligos were designed and produced by Genesky Biotechnologies Inc. (Shanghai, China).

### 2.4. Statistical Methods

The data in this study were collected into a database using EpiData 3.1 and analyzed with IBM SPSS software version 25.0 (IBM, New York, NY, USA). The measurement data had a normal distribution (mean ± standard deviation). The *t*-test was used for comparison between groups, and the count data were analyzed using the chi-square test. *P* < 0.05 was considered statistically significant. PLINK 1.07 was used for genotype distribution and haplotype analyses. Linkage disequilibrium analysis of the five adiponectin gene SNP loci was performed using SHEsis online software. The generalized multifactor dimensionality reduction (GMDR) method was used to analyze the interaction between genotypes, and T2DM environmental risk factors and the environmental risk factors affecting disease occurrence were examined using GMDR software based on a Java platform.

## 3. Results

### 3.1. Basic Characteristics of the Research Subjects

As detailed in Table 2, this study included 203 T2DM patients (case group) and 203 healthy controls (control group). The average age, BMI, WHR, fasting blood glucose, triglycerides, total cholesterol, low-density lipoprotein, and adiponectin were statistically higher in the T2DM group than in the control group. There were no differences in gender, marital status, and high-density lipoprotein between the groups. The statistically significant (*P* < 0.05) T2DM risk factors included smoking, drinking, high-fat diet, and daily exercise.

### 3.2. Correlation between the Five Loci and T2DM

All five loci obeyed the Hardy-Weinberg equilibrium (*P* > 0.5), indicating that the samples had good representativeness. Adiponectin genotype distributions in the case and control groups are detailed in Table 3.

As shown in Table 4, the association between adiponectin gene SNPs and T2DM was analyzed using two genetic models: dominant and recessive. No significant correlation was observed between the genetic patterns and T2DM.

**Figure 1:**
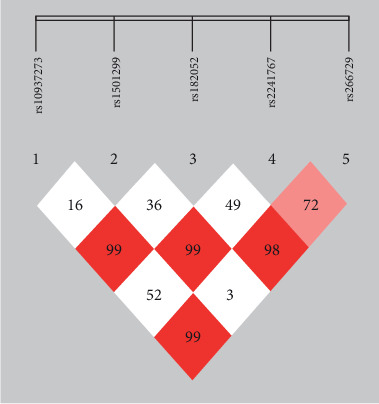
Linkage disequilibrium structure map of the five SNP loci. Note: the chain strength is proportional to color intensity.

Table 5 and Figure 1 show the linkage disequilibrium coefficient *D*′ values and correlation coefficient *r*^2^ between the five *ADIPOQ* gene SNP loci within the population. *D*′ < 0.2 indicates no linkage imbalance between two points, *D*′ > 0.5 indicates a weak linkage imbalance between two points, *D*′ > 0.8 indicates a linkage imbalance between the loci, and *D*′ = 1 signifies complete linkage disequilibrium.

Based on the frequency distribution of the five adiponectin gene loci presented in Table 6, G-G-A-A-C is a significant haplotype with an odds ratio (OR) value of 4.017 (1.201, 13.438). Under this haplotype distribution, the risk of disease was 4.017-fold higher than for the other haplotypes.

### 3.3. Gene-Environment Interactions

Table 7 details the results of the interaction analyses using the GMDR method to incorporate environmental factors into the model. The five loci interacted with hypertension history, smoking, drinking, high-fat diet, central obesity, BMI, and lack of exercise. In terms of gene-central obesity interaction, after adjusting for age, high-fat diet, hypertension, smoking, drinking, gender, BMI, and exercise, *rs1501299* and central obesity demonstrated an interaction, with a cross-consistency of 8/10 (*P* = 0.011) and a higher training and test sample accuracy (0.862, 0.835). The cross-consistency of *rs266729*, *rs182052*, and central obesity was 7/10 and *P* = 0.011, which also constitutes a better model.

## 4. Discussion

### 4.1. Analysis of T2DM Baseline Characteristics

Based on the case and control groups, there were more male than female patients and median patient age was 50–60 years. The BMI and WHR index were higher in the T2DM group than in the control group, although there was a significant difference between the two groups; BMI has become the leading risk factor for diabetes in China. Fasting blood sugar, triglycerides, total cholesterol, high-density lipoprotein, and low-density lipoprotein (LDL) were significantly different between the two groups. Total cholesterol, high-density lipoprotein, and LDL are important indicators reflecting blood lipid levels. It is well known that many factors can affect the blood lipid level of diabetic patients, as carbohydrate metabolism directly affects lipid metabolism [8, 9]. Insulin deficiency also leads to higher free fatty acid metabolism and lipid metabolism disorders [10]. Lipid abnormalities are also thought to be associated with increased vascular risk and play an important role in the pathogenesis of T2DM [8, 9]. A study in Ghana investigating blood lipids and T2DM showed that LDL and triglyceride levels increased with participant age and the number of males and also increased the risk of coronary heart disease. The parameters of all hematological examinations were statistically significant between the sexes [11].

### 4.2. History of Hypertension, Family History of Diabetes Mellitus, and T2DM

Hypertension history is an independent risk factor for T2DM and the interactions between hypertension, genetic factors, and other environmental factors are also reflected in the interaction models. As the order of hypertension and T2DM cannot be determined in a case-control study, the causal relationship between hypertension and T2DM cannot be established and it can only be identified as a risk factor. A prospective survey of diabetes and hypertension showed that >50% of diabetic patients had hypertension. Patients with hypertension and diabetes have a fourfold higher risk of cardiovascular disease than normotensive and nondiabetic controls [12].

A family history of diabetes mellitus was not a risk factor for the case group in this study. Only 17% of the patients had diabetes and there was no significant difference between the two groups. A comparison of the two groups demonstrated that smoking, drinking, and high-fat diet represent risk factors of T2DM; the OR value of high-fat diet, which had the greatest effect, was 13.59.

### 4.3. Serum Adiponectin, Obesity, and T2DM

Previous studies have shown that adiponectin, a prototypic adipocytokine, is important for the regulation of insulin resistance, as circulating levels are decreased in obesity and diseases associated with insulin resistance [13]. The ratio of adiponectin to leptin is a good indicator of adipose tissue dysfunction and an important indicator for estimating cardiac metabolic risk associated with obesity and metabolic syndrome [14]; high adiponectin levels are associated with a lower incidence of T2DM. In this study, there was no difference between the case and control groups in terms of adiponectin content owing to the limited data regarding adiponectin content. However, there were significant differences in BMI and WHR between the case and control groups.

One study conducted in Inner Mongolia in 2016 concluded that over half of the Han residents of Inner Mongolia were overweight or obese, which may increase the risk of noncommunicable diseases. The increase rate of obesity among urban Han adults in Inner Mongolia was higher than that of overweight individuals and the obese population rapidly increased [15]. The Han population in Inner Mongolia, which is affected by the local Mongolian population, consumes a greater proportion of meat in its dietary structure than in other parts of China. Moreover, drinking may also contribute to the high prevalence of overweight and obese individuals among the Han population. The drinking rate of Han men and women in the Inner Mongolia region (64.8%, 15.9%) is higher than the national level (58.3%, 8.1%) [16].

The DECODA study conducted in 2015 focused on major modifiable T2DM risk factors. They found that the etiology of T2DM is due to an interaction with ethnic-specific genetic factors. BMI and WHR have been shown to be associated with T2DM in Indian, Chinese, and Japanese individuals [17]. Therefore, although the prevention of obesity is essential for T2DM, ethnic background also requires consideration.

Exercise was a protective factor for T2DM; however, there was no significant difference between the case and control groups. It is possible that the patients had already realized that increased exercise was good for their health when they were sick. The Daqing diabetes prevention research group in China has shown that lifestyle intervention is an effective way to reduce the incidence of diabetes, but that it is insufficient. Prepregnancy planning and education regarding maternal and child health during and after pregnancy should be reinforced; intergenerational cycles initiated by epigenetic modifications resulting from adverse environmental stimuli caused by famine could fuel the T2DM epidemic [18].

### 4.4. Gene-Environment Interactions

In recent years, the association between gene-environment interactions and genetic variation characteristics has been extensively studied in T2DM [19, 20]. Traditionally, the increased number of parameters to be investigated requires a larger study population. However, a number of limitations, such as heavy computational burden (usually computationally difficult to handle) and an increase in type I and II statistical errors, can occur owing to the high sparsity of data. Ritchie et al. [21] proposed a “multifactor dimensionality reduction” method to balance case-control or discordant sibpair designs. Generalized multifactor dimensionality reduction (GMDR) is more suitable for the statistical framework of dichotomy and quantitative analysis than MDR and can adjust population phenotype covariates. Gene-environment interaction analysis showed that when adjusted for age, high-fat diet, hypertension, smoking, drinking, gender, BMI, and sport, two models were significant for the five loci-central obesity interactions.

Previous studies have shown that approximately 25% of chronic human diseases are controlled by internal genetic factors, while the other 75% depend on external factors, such as individual behavior and environment, and their interaction with internal genetic factors [22]. For example, Reddon et al. [23] defined five different patterns of gene-environment interaction according to the type of gene-environment interaction. Of these, the second mode maintains that the environment is the cause of disease; the gene itself does not directly lead to disease but can enhance the role of environmental exposure. According to the interaction results, adiponectin gene SNPs *rs1501299*, *rs266729*, and *rs182052* and central obesity have a common effect on T2DM.

In recent years, the global incidence of T2DM has significantly increased. Most cases are caused by changes in environmental and lifestyle factors. All the major risk factors of T2DM (overnutrition, low dietary fiber, sedentary lifestyle, sleep deprivation, and depression) can induce local disease or systemic low-grade inflammation, which are usually short term or mild in individuals without risk of disease; however, in individuals with T2DM, the inflammation induced by lifestyle factors is more obvious and long lasting [24]. According to a survey on the burden of diabetes mellitus and hyperglycemia in China from 1990 to 2016, high BMI and a low proportion of whole grains, nuts, and seeds in the diet were the most important risk factors for diabetes mellitus [3]. Therefore, it is necessary to generate public awareness of low-fat diets, alleviate the status of overnutrition, encourage physical exercise, and reduce the risk of T2DM. Recently, Health China Action has put forward six major actions related to health factors, including the health knowledge popularization action, reasonable diet action, national fitness action, smoking control action, mental health promotion action, and health environment promotion action, which are closely associated with the prevention and control of T2DM. The Chinese government hopes to highlight the joint efforts and roles of individuals, families, society, and the government in the prevention and control of major diseases through the implementation of these actions. Working together to promote the development of a healthy China; the overall goals are keeping the population free of illness, fewer illnesses at an early age, and improving the quality of life of the population.

Adiponectin is an important starting point for future research examining how to reduce the incidence of T2DM and mortality due to atherosclerotic diseases. Diet, exercise, and insulin sensitizers can increase adiponectin levels and reduce inflammation and insulin resistance through different mechanisms. Therefore, lifestyle changes and the use of a variety of drugs to prevent coronary atherosclerotic heart disease are recommended.

### 4.5. Research Limitations and Prospects

The study of gene-environment interactions can help elucidate the relationship between susceptibility markers and clinical effect markers and improve the accuracy and validity of SNP typing by improved multiplex ligation detection reaction (IMLDR). The interaction of adiponectin genes *rs10937273*, *rs1501299*, *rs2241767*, *rs266729*, and *rs182052* with environmental risk factors could be more clearly displayed. The sample size of this study was finite and only the association between haplotype and T2DM was determined. Furthermore, there might have been other associations that we have not discovered yet. As this was a case-control study, there were some selection biases and information biases. The environmental factors were not clearly measured but were only included in the model as categorized variables. Future studies should expand the sample size and control for the biases, more accurately measure the environmental risk factors, and increase the population size and representation in order to conduct more in-depth analyses.

## 5. Conclusions

This study analyzed the association between adiponectin gene polymorphisms and environmental factors in patients with T2DM in Hohhot, Inner Mongolia. After adjusting for age and BMI, haplotype G-G-A-A-C was susceptible to T2DM. GMDR analysis revealed that the *ADIPOQ* SNPs had interactions with central obesity. The findings of this study suggest that health education, including healthy diet and behavior patterns, should be conducted among obese individuals in Hohhot. In addition, this study may provide new insights for clinicians regarding the prevention and treatment of T2DM.

## Figures and Tables

**Table 1:** SNP information.

ID	Gene	Chromosome position	Functional consequence	Allele (major/minor)	PCR primers
rs10937273	ADIPOQ	186549695	5′-flanking	G/A	F:CACAGCCCGAAGCACTCTCAAT
					R:TTGGAATGGCTCCTCTGGTCAC

rs1501299	ADIPOQ	186571123	Intron2	G/T	F:AGATGGCACCCCTGGTGAGAAG
					R:CCTTGGAAGACCAACCCCAAAT

rs182052	ADIPOQ	186560782	Intron1	G/A	F:CCAGGCTCTCCCGTCCGTAGTA
					R:TGTGGACAGACCCTTCCACCTT

rs2241767	ADIPOQ	186571196	Intron2	A/G	F:AGATGGCACCCCTGGTGAGAAG
					R:CCTTGGAAGACCAACCCCAAAT

rs266729	ADIPOQ	186559474	5′-UTR	C/G	F:AGACACTTGCCCTGCCTCTGTC
					R:GGCCTAGAAGCAGCCTGGAGAA

**Table 2:** Comparison of demographic characteristics and biochemical indicators between the T2DM group and control group.

Parameter	Case (*n* = 203)	Control (*n* = 203)	Statistics	*P*
Age	57.08 ± 10.24	49.68 ± 10.65	7.14	<0.001^∗^
Gender (female%)	0.42	0.47	0.64	0.421
Marriage (% married)	0.98	0.99	1.01	0.807
BMI^a^ (kg·m^2^)	25.92 ± 5.30	23.53 ± 3.20	5.48	<0.001^∗^
WHR^b^	0.92 ± 0.83	0.83 ± 0.08	10.32	<0.001^∗^
Fasting blood glucose (mmol·L ^−1^)	8.68 ± 3.76	5.22 ± 1.96	11.51	<0.001^∗^
Triglyceride (mmol·L ^−1^)	2.13 ± 1.68	1.53 ± 1.14	4.12	<0.001^∗^
Total cholesterol (mmol·L ^−1^)	4.94 ± 1.30	4.56 ± 1.05	3.15	<0.001^∗^
High-density lipoprotein (mmol·L ^−1^)	1.29 ± 0.41	1.23 ± 0.42	1.52	0.128
Low-density lipoprotein (mmol·L ^−1^)	2.83 ± 1.2	2.64 ± 0.75	2.21	0.028^∗^
Adiponectin (ng·mL^−1^)	73.29 ± 16.72	77.02 ± 16.16	1.94	0.054
Family history of diabetes mellitus (%)	35 (17.24%)	31 (15.27%)	0.29	0.592
History of hypertension (%)	80 (39.41%)	7 (3.44%)	77.96	<0.001^∗^
Smoke			17.84	<0.001^∗^
Yes	76	46		
No	114	153		
Quit	13	4		
Drink			11.78	0.001^∗^
Yes	79	47		
No	124	156		
High-fat diet			131.32	<0.001^∗^
Yes	150	35		
No	53	168		
Daily exercise			3.02	0.081
Yes	117	134		
No	86	69		

Notes: ^a^body mass index, ^b^waist-to-hip ratio; serum adiponectin concentration was only measured in 162 patients and 134 controls. The case group and the control group were all Han individuals. ^∗^*P* < 0.05 probability value was considered statistically significant.

**Table 3:** Adiponectin allele and genotype distribution in the case and control groups.

dbSNPID	Genotypes (%)			*P*#	MAF (%)	HWE-P
*rs10937273*	GG	GA	AA	0.896	41.01	0.73
Case	68 (33.50)	103 (50.74)	32 (15.76)			
Control	71 (34.98)	98 (48.28)	34 (16.75)			
*rs1501299*	GG	GT	TT	0.113	25.62	0.6
Case	112 (55.17)	73 (35.96)	18 (8.87)			
Control	110 (54.19)	87 (42.86)	6 (2.96)			
*rs182052*	GG	GA	AA	0.749	42.98	0.94
Case	66 (32.51)	102 (50.25)	35 (17.24)			
Control	65 (32.02)	99 (48.77)	39 (19.21)			
*rs2241767*	AA	GA	GG	0.517	27.83	0.59
Case	106 (52.22)	84 (41.38)	13 (6.40)			
Control	108 (53.20)	74 (36.45)	21 (10.34)			
*rs266729*	CC	CG	GG	0.540	26.48	0.47
Case	110 (54.19)	82 (40.39)	10 (4.92)			
Control	105 (51.72)	83 (40.89)	15 (7.39)			

Notes: ^#^adjusted for age, BMI.

**Table 4:** Association of *ADIPOQ* genetic models with T2DM.

SNPs	Genetic models	Crude OR (95% CI)	Adjusted OR (95% CI)
*rs10937273*	Dominant model	1.068 (0.709, 1.609)	1.01 (0.537, 1.899)
	AA vs. GA+GG		
	Recessive model	0.930 (0.549, 1.576)	0.752 (0.477, 1.188)
	GA+AA vs. GG		

*rs266729*	Dominant model	0.888 (0.601, 1.312)	2.125 (0.86, 5.251)
	GG vs. CG+CC		
	Recessive model	0.649 (0.285, 1.482)	1.091 (0.707, 1.683)
	CG+GG vs. CC		

*rs182052*	Dominant model	0.977 (0.645, 1.482)	1.609 (0.915, 2.830)
	AA vs. GA+GG		
	Recessive model	0.876 (0.529, 1.451)	1.242 (0.782, 1.972)
	GA+AA vs. GG		

*rs1501299*	Dominant model	0.961 (0.650, 1.421)	0.748 (0.299, 1.869)
	TT vs. GT+GG		
	Recessive model	3.195 (1.241, 8.223)^∗^	1.454 (0.939, 2.253)
	GT+TT vs. GG		

*rs2241767*	Dominant model	1.041 (0.705, 1.536)	1.01 (0.472, 2.162)
	GG vs. GA+AA		
	Recessive model	0.59 3(0.288, 1.219)	0.973 (0.631, 1.501)
	GA+GG vs. AA		

Note: adjusted for age and BMI, ^∗^*P* < 0.05. OR: odds ratio; CI: confidence interval.

**Table 5:** Linkage disequilibrium analyses of the five SNP loci.

SNPs	rs10937273	rs1501299	rs182052	rs2241767	rs266729
*rs10937273*		0.169	0.992	0.53	1
*rs1501299*	0.007		0.361	1	0.036
*rs182052*	0.518	0.059		0.497	0.98
*rs2241767*	0.157	0.134	0.073		0.729
*rs266729*	0.252	0	0.46	0.075	

**Table 6:** Haplotype distribution of the five adiponectin gene SNPs.

Haplotypes	Case, *n* (%)	Control, *n* (%)	OR (95% CI)	Adjusted OR (95% CI)
A-G-G-G-C	79 (19.4)	85 (21)	0.960 (0.679, 1.356)	0.928 (0.592, 1.455)
A-T-G-A-C	38 (9.3)	27 (6.7)	1.511 (0.903, 2.530)	1.138 (0.600, 2.157)
G-G-A-A-C	22 (5.4)	4 (0.9)	6.723^∗^ (2.185, 20.688)	4.017^∗^ (1.201, 13.438)
G-T-A-A-G	26 (6.3)	28 (7)	0.947 (0.544, 1.648)	0.826 (0.378, 1.808)

Note: adjusted for age and BMI, ^∗^*P* < 0.05. OR: odds ratio, CI: confidence interval.

**Table 7:** Gene-environment interaction models.

Model	Training Bal	Testing Bal	CV consistency	*P*
Model 1^a^				
High-fat diet	0.794	0.795	10/10	0.001^∗^
High-fat diet/hypertension	0.819	0.82	10/10	0.001^∗^
Hypertension/high-fat diet/central obesity	0.862	0.834	10/10	0.001^∗^
*rs1501299/h*ypertension/high-fat diet/central obesity	0.862	0.835	9/10	0.001^∗^
*rs1501299/rs266729*/hypertension/high-fat diet/central obesity	0.883	0.851	9/10	0.001^∗^
Gene-central obesity interaction^b^				
Central obesity	0.64	0.639	10/10	0.001^∗^
*rs1501299*/central obesity	0.651	0.609	8/10	0.011^∗^
*rs266729*/*rs182052/c*entral obesity	0.671	0.605	7/10	0.011^∗^
Gene–high-fat diet interaction^c^				
High-fat diet	0.589	0.589	10/10	0.054
*rs10937273*/*rs1501299*	0.607	0.478	5/10	0.377
*rs1501299*/*rs182052*/high-fat diet	0.638	0.461	4/10	0.989
Gene–hypertension interaction^d^				
*rs182052*	0.565	0.474	4/10	0.828
*rs10937273*/*rs1501299*	0.599	0.434	6/10	0.945
*rs10937273*/*rs1501299*/*rs266729*	0.628	0.42	3/10	0.991
Gene–drinking interaction^e^				
*rs1501299*	0.563	0.498	5/10	0.945
*rs182052*/*rs2241767*	0.584	0.448	5/10	0.945
*rs1501299*/*rs182052*/*rs2241767*	0.612	0.462	5/10	0.945

Note: ^∗^*P* < 0.05.^a^Adjusted for age.^b^Adjusted for age, high-fat diet, hypertension, smoking, drinking, gender, BMI, and exercise.^c^Adjusted for age, central obesity, hypertension, smoking, drinking, gender, BMI, and exercise.^d^Adjusted for age, central obesity, high-fat diet, smoking, drinking, gender, BMI, and exercise.^e^Adjusted for age, central obesity, high-fat diet, hypertension, smoking, gender, BMI, and exercise.

## Data Availability

The data used to support the findings of this study are available from the corresponding author upon request.
